# Rhabdoviruses, Antiviral Defense, and SUMO Pathway

**DOI:** 10.3390/v10120686

**Published:** 2018-12-03

**Authors:** Faten El Asmi, Carlos Eduardo Brantis-de-Carvalho, Danielle Blondel, Mounira K. Chelbi-Alix

**Affiliations:** 1 INSERM UMR-S 1124, Université Paris Descartes, 75006 Paris, France; elasmi.faten@gmail.com (F.E.A.); kdubrantis@icloud.com (C.E.B.-d.-C.); 2 Institute for Integrative Biology of the Cell (I2BC), Université Paris-Saclay, CEA, CNRS UMR 9198, Université Paris-Sud, 91190 Gif-sur-Yvette, France; danielle.blondel@i2bc.paris-saclay.fr

**Keywords:** interferon, rabies virus, vesicular stomatitis virus, IFN, SUMO, MxA, PKR

## Abstract

Small Ubiquitin-like MOdifier (SUMO) conjugation to proteins has essential roles in several processes including localization, stability, and function of several players implicated in intrinsic and innate immunity. In human, five paralogs of SUMO are known of which three are ubiquitously expressed (SUMO1, 2, and 3). Infection by rhabdoviruses triggers cellular responses through the activation of pattern recognition receptors, which leads to the production and secretion of interferon. This review will focus on the effects of the stable expression of the different SUMO paralogs or Ubc9 depletion on rhabdoviruses-induced interferon production and interferon signaling pathways as well as on the expression and functions of restriction factors conferring the resistance to rhabdoviruses.

## 1. Introduction

### 1.1. SUMO Pathway

The post-translational modification with the Small Ubiquitin-like MOdifier (SUMO) is a reversible reaction that controls the localization, the stabilization, and the functional state of its protein targets leading to the regulation of several biological processes including gene transcription, PML nuclear body formation, innate immunity modulation, and an antiviral defense [1,2,3,4,5,6]. In humans, three SUMO paralogs (SUMO1, SUMO2, and SUMO3) are ubiquitously expressed and can act as protein modifiers. SUMO2 and SUMO3 are highly homologous proteins, which shares 95% sequence identity and collectively referred to as SUMO2/3, but they only share 50% amino acid identity with SUMO1 [7], which correlates with differential functions of these paralogs [3,8,9].

**Figure 1:**
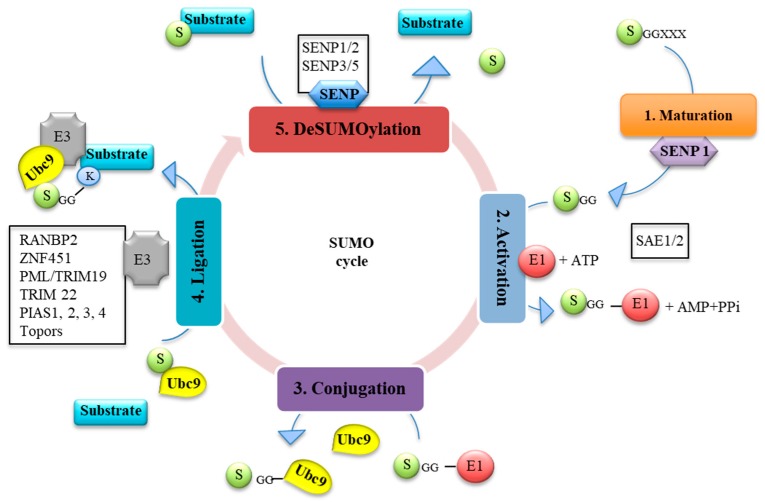
The SUMO cycle. SUMOylation is processed in five major steps: 1. Maturation: The immature form of SUMO is processed by specific proteases (SENPs) (to expose its C terminal diglycine (GG) motif (X: any amino acid). 2. Activation: in an ATP consuming reaction, the E1 activating enzyme (SAE1/2) activates the matured SUMO to form a high energy thioester bond. 3. Conjugation: SUMO is then transferred to the E2 conjugating enzyme (Ubc9) and form a thioester bond. 4. Ligation: SUMO is conjugated to its substrate with the help of an E3 ligase and 5. DeSUMOylation: SUMO can be removed from its target substrate by SENPs and engage in a new cycle.

SUMO proteins interact with substrates in two ways. First, SUMO can be covalently conjugated at the carboxyl terminus group of SUMO with the amino group of a lysine residue on the substrate. This modification is named SUMOylation. In this process, SUMO paralogs are expressed as immature precursors and require an initial maturation step where SUMO is cleaved by SUMO specific proteases (SENPs) to expose its C-terminal di-glycine (GG) motif, which is mandatory for the conjugation. The activation process is then initiated with the SUMO activating enzyme (E1) composed of the SAE1/SAE2 (AOS1/UBA2) subunits, which interacts with SUMO in the presence of ATP to form a high-energy thioester bond. E1 recognizes the SUMO conjugating enzyme (E2) and Ubc9 and promotes the SUMO transfer to the E2 enzyme. The final conjugation to the target protein can be conducted by an E3 ligase. SENPs can also reverse the SUMOylation machinery by cleaving the isopeptide bond between SUMO and its substrate, which delivers free mature SUMO paralogs to re-conjugation or degradation and, thus, maintains a certain balance within the cell. SUMO conjugation to its substrates occurs via an acceptor lysine (K) within a consensus motif (ψKxE) where (ψ) is a hydrophobic amino acid and (x) a random amino acid. SUMO2 and 3 contain the consensus motif, which enables them to form poly SUMO chains [10,11,12] while SUMO1 lacks the consensus site, is consequently unable to form poly chains, and acts as a poly SUMO chain terminator [10,13]. As stated, the covalent linkage to substrate involves four enzymes including a single E1-activating enzyme (SAE1/SAE2), a unique E2-conjugating enzyme (Ubc9), and several E3 ligases, which leads to the final conjugation [14] including the SP-RING domain (Siz/PIAS) family, RANBP2 [15], ZNF451 [16], Topors [17], and other TRIM family members among which are TRIM19/ProMyelocytic Leukemia (PML) [18] and TRIM22 [19] (Figure 1). Second, all SUMO paralogs interact in non-covalent manner through the SUMO-interacting motifs (SIMs) affecting protein activity without being covalently attached [5]. The short core sequence of hydrophobic amino acids (V/I/L)X(V/I/L)(V/I/L) was reported to be the minimal motif needed in target proteins for a SUMO interaction [20].

### 1.2. Regulation of SUMO and SUMOylation by Viruses and IFN

SUMOylation has emerged as a key post-translational modification that can be used by the host [2,9] or the viruses to alter antiviral responses and viral replication [21,22]. During infection, viruses can manipulate the SUMOylation process to ensure viral persistence within the host. Members of several viral families have been shown to modulate SUMOylation including papillomaviruses, adenoviruses, herpesviruses, orthomyxoviruses, filoviruses, and picornaviruses [21,22,23]. Although it is unknown whether infection with rhabdoviruses modulates global cellular SUMOylation, infection with a Vesicular Stomatitis Virus (VSV) promotes the SUMOylation of several cellular proteins such as the Interferon Regulatory Factor (IRF) 3 and IRF7 [24] as well as p53 [25], phosphatase, the tensin homolog deleted for chromosome 10 (PTEN) [26], and the double-stranded RNA-dependent protein kinase (PKR) [27].

Recently, it has been reported that interferon (IFN) treatment increases the global cellular SUMOylation and requires the presence of the SUMO E3 ligase PML, which are the organizer of PML nuclear bodies (NBs) [28]. Introducing each of the human PML isoform (PMLI to PMLVII) in PML-negative cells has demonstrated that increased SUMOylation in response to IFN is orchestrated by PMLIII and PMLIV isoforms. In addition, PML is required for IFN-induced Ubc9 transfer to the nuclear matrix where both Ubc9 and PML are found co-localizing within PML NBs [28]. Several SUMO sites have been shown by large-scale proteomic experiments to be regulated upon IFN stimulation including K49 from Ubc9 [28]. Ubc9 SUMOylation on K49 is required for its localization within PML NBs [29]. These findings suggest that PML and Ubc9 act in a cooperative manner to enhance cellular SUMOylation upon IFNα stimulation, which further demonstrates that PML NBs are a hub for protein SUMOylation.

The SUMOylation process begins with the transcription of the *SUMO* genes. To date, only the promoter of *SUMO1* has been identified and reported to contain NF-κB, FOXP3, p53, and TCF-4E binding sites [30]. It has been shown that the influenza virus infection increases SUMO levels without enhancing *SUMO* mRNA transcripts [23]. Future studies will reveal whether SUMO promoters could be activated upon other viral infections. Additionally, IFN treatment without altering *SUMO1* mRNA levels enhances unconjugated SUMO1 levels through an miRNA-based mechanism involving the Lin28/let-7 axis, which contributes to the antiviral effect of IFN [31]. Taken together, these results show that *SUMO1* is not directly induced in response to IFN. Its promoter does not contain the IFN response elements and, therefore, *SUMO* is not an ISG.

**Figure 2:**
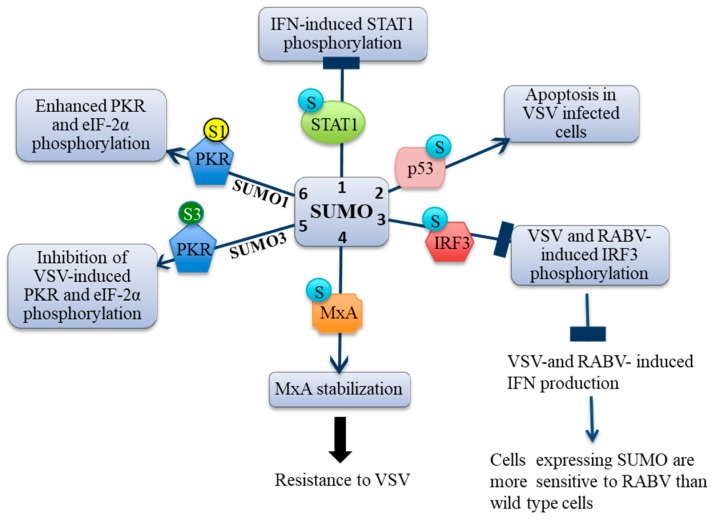
Effect of SUMO on IFN pathways. In SUMO1-expressing and SUMO3-expressing cells: 1. STAT1 activation by IFN stimuli is inhibited by its SUMOylation [3]. 2. p53 SUMOylation is required for the induction of apoptosis in VSV infected cells [25]. 3. IRF3 is SUMOylated, which causes an inhibition of VSV-induced and RABV-induced IFN synthesis. This results in a higher RABV replication [2]. 4. SUMOylation of MxA in SUMO-expressing cells leads to MxA stabilization and to increased resistance to VSV infection [2]. 5, 6. SUMO1 (S1) and SUMO3 (S3) have different effects on PKR and eIF-2α activation [9].

The actions of SUMO on IFN pathways are both dynamic and complex. In this review, we will focus on the effect of stable expression of the different SUMO paralogs or Ubc9 depletion on IFN signaling, on IFN synthesis, and restriction factors in cells infected with rhabdoviruses. SUMO alters IFN responses at different steps that are summarized in Figure 2. SUMO alters signaling of type I and II IFNs [3], rhabdoviruses-induced IFN production [2,24], and restriction factors playing key roles in conferring resistance to these viruses [2,9,27].

## 2. Rhabdoviruses

Rhabdoviruses (order Mononegavirales) constitute a family of viruses having a particularly broad host range among a great diversity of organisms including plants, insects, crustaceans, fishes, reptiles, and mammals. They are associated with significant pathologies in humans and livestock [32]. The prototypes of this family are vesicular stomatitis virus (VSV), which is a member of the *Vesiculovirus* genus, and rabies virus (RABV), which is a member of the *Lyssavirus* genus.

VSV infects horses, cattle, pigs, and a range of other mammals and their insect vectors are natural hosts of VSV [32]. The infections of livestock are non-lethal but can cause severe foot-and mouth-like disease. Human VSV infections are generally asymptomatic and limited to agricultural and laboratory workers.

RABV is a neurotropic virus that causes acute encephalitis in humans and animals with almost always lethal outcomes. Human rabies is a zoonosis, which still accounts for ~60,000 deaths per year worldwide even though effective vaccines are available.

Rhabdoviruses have a bullet-like shape, which is rounded at one end and flat at the other, with a diameter of ~75 nm and a length of ~180 nm. The genome is a negative sense, single stranded RNA molecule of approximately 12 kb flanked on both sides by a non-coding leader and trailer RNA regions. It comprises only five genes that are common to all members of the family, which, starting from the 3’ terminus, successively encode the nucleoprotein (N), the phosphoprotein (P), the matrix protein (M), the glycoprotein (G), and the large subunit of the RNA-dependent RNA polymerase (L). The viral RNA is tightly associated with the N protein to form a helical nucleocapsid. The nucleocapsid interacts with the viral polymerase complex composed of L and its non-enzymatic cofactor P to form the ribonucleoprotein (RNP). The RNP is enwrapped by a lipid bilayer, which is derived from a host cell membrane during the budding process. The matrix protein (M) is located beneath the viral membrane and bridges the RNP and the lipid bilayer, which contains a single transmembrane glycoprotein (G) that is involved in viral entry.

**Figure 3:**
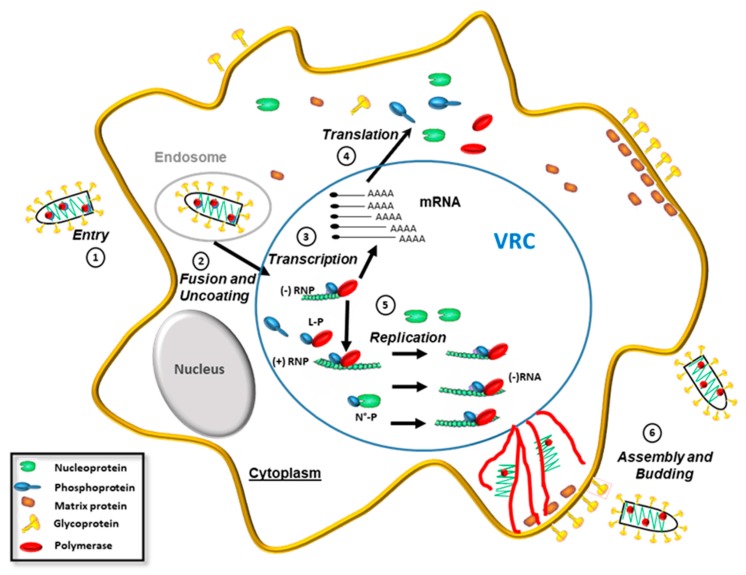
Rhabdoviruses life cycle. Several steps can be observed in the cytoplasm: 1. entry phase involving the binding of viral particles to receptors. 2. Endocytosis followed by membrane fusion and RNPs release in the cytosol. 3. Transcription of viral mRNA. 4. Translation of viral mRNAs by the cell machinery. 5. Replication of the viral genome and 6. transport of viral RNP by microtubules to membrane virus assembly and progeny virus budding. Viral transcription and replication occur in viral replication compartments (VRC), which are phase-separated liquid compartments.

The cellular cycle of rhabdoviruses is entirely cytoplasmic (Figure 3). After binding to a receptor, the viral particle enters the cell via the endocytic pathway [33,34,35]. Thereafter, the acidic environment within early endosomes induces a conformational change of G that catalyzes fusion of the viral envelope with the endosomal membrane [36]. This results in the cytoplasmic release of the negative-sense RNP, which constitutes the template for viral transcription and replication by the L-P complex. These processes take place within viral inclusions [37,38], which, in the case of RABV, are termed Negri bodies. For both RABV and VSV, these viral replication compartments (VRC) have the properties of liquid-like compartments that form by phase separation [39,40]. Transcription starts at the 3′ end of the genome RNA and results in the synthesis of a positive, uncapped, and short leader RNA and five capped, poly-adenylated mRNAs encoding the five viral proteins. During this process, the L-P complex scans across the gene junctions after the addition of the poly A-tail via reiterative copying of the poly U tract at the end of each gene. When the polymerase reaches a consensus gene start at the next gene, the transcription is reinitiated. Viral mRNAs are then translated by the host cell translation machinery and provide a source of the N protein necessary to encapsulate the nascent RNA. This results in the switch of the activity of the polymerase complex from transcription to replication to produce RNPs containing full-length antigenomic RNA (positive sense), which, in turn, serve as templates for the synthesis of genomic RNA (negative sense) [41]. In the VRC, the neo-synthesized genomic RNPs then serve as templates for additional rounds of transcription and/or replication [41]. They can also be ejected from the VRC, which is transported by microtubule networks to membranes [39] enriched in both M and G proteins. Interactions between RNP, M, and G then drive the assembly of viral particles and subsequent budding.

## 3. Effects of SUMO on IFN Signaling and Production

SUMOylation targets many cellular proteins implicated in IFN synthesis, the IFN JAK/STAT signaling pathway, and IFN-stimulated gene (ISG) restriction factors playing key roles in antiviral defense [1].

### 3.1. SUMO Inhibits IFN-Induced STAT1 Activation

Three classes of IFNs have been identified and are designated as types I to III [42,43]. The human type I IFN comprises 17 distinct proteins, which are mainly represented by IFNα and IFNβ. IFNγ is the unique member of type II IFN family and type III IFN is composed of four homologous proteins (IFNλ1–4). Although type I and type III IFNs bind to different cell receptors, they activate the same signal transduction pathway resulting in the increase of ISGs, which are the products that mediate IFN-induced biological responses [44].

Type I and III IFNs exert their function via the activation of JAK kinases namely Tyk2 and JAK1 and the phosphorylation of the STAT1 and STAT2 transcription factors that form a heterodimer. This heterodimer forms a complex with IRF9 named the IFN-stimulated gene factor 3 (ISGF3), which moves into the nucleus where it binds to the IFN-stimulated response element (ISRE) sequence present in the promoters of ISGs.

IFNγ activates JAK1 and JAK2 resulting in the phosphorylation of STAT1, which is the key transcription factor of IFNγ signaling. Phosphorylated STAT1 homodimerizes and forms a complex named gamma-activated factor (GAF) that migrates to the nucleus and binds to the gamma-activated site (GAS) located at the promoters of ISGs.

In cells stably expressing SUMO1 or SUMO3, STAT1 is SUMOylated and IFNα- or IFNγ-induced STAT1 phosphorylation is highly inhibited (Figure 2). In a converse experiment, depletion of Ubc9, which is the unique E2-conjugating enzyme for SUMOylation in wild type cells results in a decrease of the level of SUMO2/3-modifed proteins and in a higher level of STAT1 phosphorylation in response to IFNγ or IFNα [3]. The stable expression of SUMO1 or SUMO3 in human cell lines inhibits IFNγ-induced STAT1 activation, transcription of ISGs, and, therefore, impairs downstream events [3]. In contrast, although overexpression of SUMO1 or SUMO3 decreases STAT1 activation in response to IFNα, it does not alter STAT2 phosphorylation, binding to ISRE, or the transcriptional response [3]. These results further demonstrate that, while STAT1 cooperativity is essential to the IFNγ response, it is dispensable for IFNα signaling. Emerging reports show that, in response to IFNα, STAT2 forms independently from STAT1, a complex with the ISRE binding IRF9 and mediates ISG expression [45,46,47,48], which supports the existence of alternative STAT2 signaling pathways that are independent from STAT1.

### 3.2. SUMO Inhibits VSV-Induced and RABV-Induced IFN

Several reports showed that post-translational modifications such as ubiquitination and SUMOylation are key regulators of virus-induced IFN synthesis [1]. The activation of the IFN production upon viral infection is initiated by detecting viral RNA using the cytosolic helicases retinoic acid-inducible gene I (RIG-I) and the melanoma differentiation-associated gene 5 (MDA5). SUMO has been reported to positively regulate innate immunity since SUMOylation of MDA5 and RIG-I has been associated with an increase of type I IFN production [49,50]. In contrast, the SUMOylation of the DNA binding IRF3 and IRF7, which are the key mediators of IFN synthesis that are essential for antiviral innate immunity, reduces VSV-induced IFN synthesis. They have been shown in cells transfected with Flag-IRF3 or Flag-IRF7 to be covalently conjugated to SUMO1, SUMO2, and SUMO3. Their SUMOylation is markedly increased following VSV infection [24]. Accordingly, compared to the wild type IRFs, cells expressing IRF3 or IRF7 mutants defective in SUMOylation have higher levels of *IFNα* and *IFNβ* mRNA expression after VSV infection, which suggests that SUMOylation inhibits virus-induced IFN production [24].

More recently, it has been reported that stable expression of different SUMO paralogs in human cells highly decreases VSV-induced IFN production. *IFNα*, *IFNβ*, *IFNλ1*, *IFNλ2/λ3*, and *IFNγ* mRNAs are induced in HeLa cells post-VSV infection while no significant increase in the expression of these mRNAs is observed in infected HeLa cells expressing SUMO1 or SUMO3 [2], which suggests that SUMO inhibits the synthesis of type I, II, and III IFNs upon VSV infection. SUMO3 expression increases IRF3 SUMOylation and also decreases RABV-induced IFN-β. This inhibition of IFN synthesis is due to a lower level of phosphorylated IRF3 in RABV-infected SUMO3 cells when compared to that in infected wild type cells. As expected, in SUMO-expressing cells, the inhibition of RABV-induced IFN renders cells more sensitive to this virus [2]. Intriguingly, although VSV-induced IFN production is inhibited, SUMO-expressing cells are resistant to this virus [2] due to the stabilization of the anti-VSV restriction factor MxA (for MyXovirus resistance) (see below).

Taken together, these reports show that increasing the SUMOylation levels through overexpression and inhibiting through Ubc9 depletion reduces and boosts IFN synthesis in infected cells. This suggests that SUMO negatively regulates the innate immune response by decreasing IFN production.

## 4. Effect of SUMO on Restriction Factors Conferring Resistance to Rhabdoviruses

### 4.1. Restriction Factors Conferring Resistance to Rhabdoviruses

The establishment of an antiviral state in cells is the defining function of IFNs. Any stage in viral replication may be a target for inhibition by IFNs via ISG restriction factors (reviewed in Reference [51]). Several restriction factors confer VSV resistance by targeting different steps of VSV replication [51] (Table 1).

The IFN-inducible transmembrane (IFITM) proteins inhibit entry [52]. Cholesterol-25-hydroxylase (Ch25h) targets fusion and uncoating [53], MxA inhibits primary transcription [54,55], PML [58,59] and ISG20 [56] proteins inhibit the secondary transcription, PKR [60] inhibits viral translation, and Tetherin prevents release of virions from the cell [52]. The IFN-induced proteins with tetratricopeptide repeats (IFIT2 and IFIT3) are also implicated in conferring resistance to VSV since VSV production is higher in their absence [61,62,64]. In the case of RABV, expression of one PML isoform, PMLIV, confers resistance to this virus by inhibiting secondary transcription [57] and, more recently, it has been reported that depletion of IFIT2 in neuroblastoma cells results in an increase of RABV replication [63]. In addition, the Guanylate Binding Protein 1 (GBP1), which belongs, like MxA, to the dynamin superfamily of large GTPases, inhibits VSV production. However, the mechanism is still unknown [65]. The IFN-induced tumor suppressor p53 is also an anti-VSV effector since more VSV is produced in the absence of p53 [67]. PTEN is a tumor suppressor gene that has been shown to be implicated in innate immunity [68] and to inhibit the replication of VSV in mice [69]. Whether PTEN is an ISG restriction factor is unknown.

### 4.2. VSV Restriction Factors and SUMO

Among the restriction factors conferring resistance to VSV, some have been shown to be covalently modified by SUMO and require this modification to inhibit VSV, namely PML [58], p53 [25], PTEN [26], MxA [2], and PKR [27].

PML is the organizer of nuclear matrix-associated structures named nuclear bodies (NBs). PML is mainly covalently conjugated to SUMO at three sites K65, K160, and K490 [70] and this modification is required for PML NB functions and for the capacity of PML to interact with other partners [1]. PML was shown to inhibit VSV in two ways [58]. It can exert an intrinsic activity inhibiting VSV secondary transcription and can also enhance the innate immune activity by increasing VSV-induced IFNβ synthesis. In both cases, the SUMOylation of PML is required [58]. In addition, the SUMOylation of PML and p53 is required to confer resistance to RABV [57] and VSV [25], respectively. Additionally, PTEN SUMOylation contributes to the control of VSV [26]. VSV infection was shown to induce PTEN SUMOylation and its translocation to the cell membrane [26]. Recent findings show that stable expression of the different paralogs of SUMO has consequences on the functions of the restriction factors MxA and PKR (see below).

### 4.3. SUMO Confers an Intrinsic Resistance to VSV and Not to RABV

The conjugation of SUMO3 to IRF3 reduces both its activation and IFN production upon RABV infection, which renders the cells more susceptible to this viral infection. In contrast, although stable expression of the different SUMO paralogs in human cells inhibits VSV-induced IFN synthesis, it confers an intrinsic resistance to VSV infection. SUMO does not affect VSV entry but blocks primary mRNA synthesis, which results in a reduction of viral production and cell protection from VSV-induced cell lysis. SUMO [2] like MxA [51] inhibits VSV primary transcription (Table 1).

### 4.4. MxA Is Conjugated to SUMO

Mx proteins are evolutionarily conserved dynamin-like large GTPases involved in viral resistance triggered by type I and III IFNs [71]. MxA is organized in three domains: an N-terminal GTP-binding domain (GTPase), a central interactive domain (CID), and a C-terminal GTPase effector domain (GED) [72]. MxA is also known to self-assemble into oligomers that have a stabilizing effect on the protein [73] and are necessary to inhibit several different types of viruses by blocking the early steps of their replication cycle [71,74]. Ectopic expression of human MxA confers resistance to VSV by inhibiting primary transcription [54,55] but does not alter RABV replication [2].

### 4.5. MxA Mediates SUMO-Induced VSV Resistance

When screened for binding partners, using the yeast two-hybrid system MxA was shown to have 27 putative ligands with many of them related to the SUMOylation machinery or known to be SUMOylated proteins. MxA oligomerization capacity is important for its interaction with SUMO and Ubc9 [75]. Accordingly, the monomeric mutant MxA (L612K) has a reduced interaction with SUMO and Ubc9 [75] and is rapidly degraded in cells when compared to wild type MxA [73] demonstrating that self-assembly of MxA protein is critical for protein stability. In addition, MxA is SUMOylated at a unique lysine 48 (K48) SUMOylation site [75]. The SUMOylation-deficient mutant of MxA (MxA-K48R) retains its capacity to oligomerize and to inhibit VSV. However, this occurs with a lower efficiency than does the MxA wild type [75].

As mentioned above, the expression of the different SUMO paralogs inhibits VSV and RABV-induced IFN and this renders cells more sensitive to RABV but intriguingly SUMO-expressing cells are resistant to VSV infection due to the high inhibition of VSV primary transcription [2]. Among the restriction factors conferring resistance to VSV, only MxA protein inhibits the viral primary transcription [51] (Table 1).

The MxA protein was found to be highly stabilized in SUMO-expressing cells through an increased MxA oligomerization state, which suggests that SUMO may play a role in protecting MxA from degradation, which provides a stable intracellular pool of MxA able to protect cells from viral infection [2]. It has been shown that Ubc9 depletion in SUMO-expressing cells decreases the level of SUMO-conjugated proteins but does not reduce SUMO-induced VSV resistance because MxA protein levels are maintained at stable levels in SUMO expressing cells depleted for Ubc9 [2].

The key role of MxA in mediating SUMO-induced VSV inhibition is demonstrated by the fact that MxA depletion abolishes SUMO-induced VSV resistance [2]. It should be noted that MxA overexpression does not alter RABV infection. Therefore, even though MxA is stabilized, this has no consequences on RABV infection [2].

Further investigations are needed to determine whether MxA stabilization in SUMO-expressing cells could also confer resistance to other viruses known to be inhibited by MxA.

## 5. SUMO Paralogs Differentially Alter PKR Activation

PKR is among the ISG products with important biological functions [76,77]. PKR, which is a 68 kDa serine/threonine kinase, is ubiquitous and constitutively expressed. PKR is induced in an inactive form by IFN and activated by autophosphorylation upon binding to viral double-stranded (ds) RNA.

Activated PKR phosphorylates several substrates with the most studied being the α subunit of the protein synthesis initiation factor eIF-2α [78], which results in an inhibition of protein synthesis and viral propagation [79]. PKR contains an N-terminal double stranded RNA binding domain and a C-terminal kinase domain (residues 258–551). In addition to dsRNA, PKR can be activated by heparin [80], PKR activating protein [81], or ISG15 [82].

In addition to phosphorylation, PKR was found to be ISGylated [82] and SUMOylated [27]. PKR is conjugated to SUMO at lysine residues Lys-60 and Lys-150, which is located at the dsRNA binding domain of PKR, and Lys-440, which is located at the C-terminal domain of the protein [27]. Moreover, mutation of the lysine residues K60-K150-K440 SUMOylation sites in PKR abolishes its ability to inhibit protein synthesis in response to dsRNA and significantly reduces its proapoptotic and anti-VSV activities [27], which suggests a critical role of SUMO in the PKR activity.

More recently, it has been shown that ectopic expression of SUMO1 and SUMO3 has differential effects on PKR activation (phosphorylation). Ectopic SUMO1 expression alone in human cells is able to activate PKR resulting in eIF-2α phosphorylation with enhanced phosphorylation of PKR and eIF-2α when cells were infected with VSV [9]. In contrast, Ubc9 depletion in HEK293 cells reduces the phosphorylation of PKR and eIF-2α in response to VSV infection [27]. Furthermore, the fact that the expression of SUMO1 is able to activate PKR in the absence of viral infection results in a gain of PKR activity and suggests a novel mechanism for PKR activation. PKR ISGylation at K69 and K159 by ISG15, which is another ubiquitin-like modifier, results in PKR and eIF-2α phosphorylation in the absence of viral infection [82]. At the opposite end, SUMO3 expression by itself does not alter PKR activation but reduces VSV-induced PKR and eIF-2α activation, which counteracts the PKR function [9]. These findings shed a new light on the differential effects of SUMO paralogs on PKR activation.

## 6. Conclusions

SUMOylation of a large number of signaling proteins and restriction factors provide vital mechanisms for the regulation of intrinsic and innate immune responses. Significant advances have been made over the last 20 years in the field of SUMO. However, we are only beginning to understand the impact of this post-translational modification in an antiviral defense. We have reviewed the effect of stable expression of SUMO on VSV and RABV-induced IFN production and the restriction factors conferring resistance to VSV such as PKR and MxA. It will be interesting to determine whether other ISG restriction factors are, like MxA, stabilized by SUMO and, thereby, could contribute to confer resistance to RNA or DNA viruses. In addition, emerging reports show that the SUMO paralogs could have different functions. SUMO1 activates PKR while SUMO3 blocks its activation upon VSV infection. Understanding the effects of the different SUMO paralogs on the IFN pathway and ISG restriction factors may help discover novel mechanisms in an antiviral defense. The role of the SUMO pathway in an antiviral defense can be investigated through the formation of VRC such as Negri bodies. Such liquid compartments may be a way for the virus, in addition to the concentrate of the replication machinery, to evade detection by innate immunity. Alternatively, the cells may have evolved a mechanism, which allows the sensing of such structures and/or their destabilization. Proteomic experiments are planned to study the interaction between VRC and cellular components of the innate immunity such as proteins belonging to the IFN pathway. Once such proteins associated with VRC are identified, their role will be analyzed and, thereafter, the impact of their SUMOylation could be investigated on the formation, the size, and the dynamics of VRC. The formation and composition of PML NBs, which are also liquid organelles, are controlled by PML SUMOylation [83]. In addition, PML has been reported to be a SUMO ligase [18] that is essential for IFN-enhanced global cellular SUMOylation [28]. Understanding the effects of SUMOylation on the IFN pathway, ISG restriction factors and on liquid organelles formation could help discover new ways for developing antiviral therapies.

## Figures and Tables

**Table 1:** Steps of the VSV life cycle inhibited by restriction factors and SUMO.

Restriction Factors and SUMO Inhibiting VSV	Inhibition	References
IFITM3	Entry	[52]
Ch25h	Fusion and uncoating	[53]
$\begin{array}{c} \mathrm{MxA}\;{}^{1}\\ \mathrm{SUMO}\;{}^{2} \end{array}\}$	Primary transcription	[54,55]
		[2]
$\begin{array}{c} \mathrm{ISG}20\\ \mathrm{PML}\;{}^{3} \end{array}\}$	Secondary transcription	[56]
		[57,58,59]
PKR	Translation	[60]
IFIT2 ^4^	Replication	[61,62,63]
IFIT3	Production	[64]
Tetherin	Assembly and budding	[52]
GBP1	Production	[65]
p53	Production	[25,66]

^1^ Expression of MxA does not confer resistance to RABV [2]. ^2^ Expression of SUMO highly stabilizes MxA protein and depletion of MxA abolishes SUMO-induced anti-VSV activity [2]. ^3^ Expression of one PML isoform, PMLIV, inhibits RABV at the level of secondary transcription [57]. ^4^ IFIT2 inhibits RABV replication [63] and VSV production [61,62].
